# A new black nightshade (Morelloid clade, *Solanum*, Solanaceae) from the caatinga biome of north-eastern Brazil with a key to Brazilian morelloids

**DOI:** 10.3897/phytokeys.108.27254

**Published:** 2018-08-13

**Authors:** Sandra Knapp, Tiina Särkinen

**Affiliations:** 1 Department of Life Sciences, Natural History Museum, Cromwell Road, London SW7 5BD, UK The Natural History Museum London United Kingdom; 2 Royal Botanic Garden Edinburgh, 20A Inverleith Row, EH3 5LR Edinburgh, UK Royal Botanic Garden Edinburgh United Kingdom

**Keywords:** Brazil, dry forests, endemism, identification key, new species, *
Solanum
*, Solanaceae, weeds

## Abstract

*Solanumcaatingae***sp. nov.** is described from the arid caatinga biome of north-eastern Brazil. It is known from only a few specimens, but these were found amongst the many sheets of the widespread circumtropical weed *S.americanum* Mill.; it is possible that more will be found once its distinct nature has been recognised. It differs from *S.americanum* and all other herbaceous black nightshades known in Brazil, in its combination of glandular pubescence and shiny black fruit with small spreading sepals. The description of *S.caatingae* brings the number of morelloid solanums in Brazil to seven and a key is provided for their identification.

## Introduction

*Solanum* L. is one of the most species-rich vascular plant genera in South America (Jørgensen et al. 2011; Ulloa Ulloa et al. 2017) and, within the continent, the tropical Andes represent one of the main centres of species diversity for *Solanum* for both spiny (Leptostemonum clade, see Stern et al. 2011) and non-spiny clades (Weese and Bohs 2007; Särkinen et al. 2013b). Significant diversity is also found in dry regions such as the Atacama desert (Regmandra clade, Bennett 2008) and seasonally dry tropical forests (Cyphomandropsis clade, Bohs 2001; Dulcamaroid clade, Knapp 2013; Geminata clade, Knapp 2002a, 2008; section GonatotrichumStern et al., 2013;sectionErythrotrichum, Agra 2008; Elaeagnifolium clade, Knapp et al. 2017). These dry habitats, however, are less species-rich, but also less well-explored (Sobral and Stehmann 2009; Sousa-Baena et al. 2014), than the more humid forests of the Andes and south-eastern Brazil, which have been considered the foci of diversity in the genus (Knapp 2002b).

Within *Solanum*, the Morelloid clade is a group of ca. 75 species most of which are endemic to the tropical Andes (Bohs 2005; Särkinen et al. 2015c). The clade includes five major groups traditionally recognised at the sectional level (sections *Solanum*, *Campanulisolanum* Bitter, *Parasolanum* A.Child pro parte, *Chamasarachidium* Bitter and *Episarcophyllum* Bitter), which are in the process of re-circumscription based on molecular results (Särkinen et al. 2015c). The black nightshade group (sectionSolanum sensu D’Arcy 1972; see Särkinen et al. 2018) is the largest of these with ca. 52 species and ca. 580 published names and is the only group to occur outside of the Americas. Black nightshades are distinguished by their herbaceous to sub-shrubby habit, stems that sometimes bear spinescent processes (not true prickles, see Edmonds 1972; Särkinen et al. 2015c), inflorescences usually positioned along the internodes, small flowers and fruits and the usual possession of stone cells in the fruits (Bitter 1911), which appear as small, seed-like structures that are usually white and spherical rather than flattened and brown or yellowish-brown like the seeds. These stone cells are derived from accretions of sclerenchyma in the mesocarp (Bitter 1911, 1914; Danert 1969). Although some studies have been undertaken to clarify the taxonomy of the Old World and North American species of the Morelloid group (Edmonds 1977, 1978; Schilling 1981; Särkinen et al. 2018), a monographic study is needed to aid species identification and to clarify synonymy, especially in Andean South America where most of the species diversity is found (Edmonds 1972; Barboza et al. 2013) and where the Morelloid clade is amongst the most diverse groups of *Solanum*. The weedy nature of many of the common species of the group means that distinct taxa are often identified as common species and collections are often not made because botanists consider these plants uninteresting weeds (see Särkinen et al. 2013a for a typical case).

Recent taxonomic work, focusing on delivering a global monographic treatment of the Morelloid clade, has resulted in the description of various new species from the tropical Andes (Särkinen et al. 2013a, 2015a, 2015b; Särkinen and Knapp 2016). Unlike for many groups of solanums (e.g. Giacomin and Stehmann 2014; Knapp et al. 2015; Gouvêa and Stehmann 2016), Brazil is not a centre of diversity for the Morelloid clade, but in work undertaken revising these species we have encountered specimens from the poorly explored caatingas of north-eastern Brazil that do not correspond to any of the currently recognised species from the region. We describe this taxon here and provide a key to all morelloid species occurring in Brazil and current documentation of our knowledge of specimens of these taxa in Brazil.

## Materials and methods

The description of *S.caatingae* is based on examination of herbarium specimens from CEPEC, HUEFS, RB and W (acronyms follow Index Herbariorum; http://sweetgum.nybg.org/science/ih/). Specimens of this species may be found identified as *S.americanum* in other herbaria, but in our extensive work on the morelloid solanums in European, American and Latin American herbaria (see Särkinen et al. 2018), we have failed to find collections other than those cited here. Specimens (1,241 in total) included in the Suppl. material 1 are those morelloids occurring in Brazil for which we have examined material from 68 herbaria worldwide; many of these are not yet georeferenced but will be as we complete the monographic treatment of this group for South America (e.g. see Särkinen et al. 2018). The specimen data are included here to assist herbarium curators with species identification.

Specimens with coordinates were mapped directly and those lacking coordinates were located using Google Earth and gazetteers. The Extent of Occurrence (EOO) and Area of Occupancy (AOO) were calculated using GeoCat (www.geocat.kew.org) with a 2 km cell width for AOO calculation. The preliminary conservation status was assessed using the IUCN (2017) criteria based on the GeoCat analyses (Bachman et al. 2011) combined with field knowledge. All specimens are cited in the text and full data is provided in the supplemental file and on the NHM Data Portal (https://doi.org/10.5519/0034287).

## Taxonomic treatment

### Solanum caatingae

Taxon classificationPlantaeSolanalesSolanaceae

S.Knapp & Särkinen sp. nov.

urn:lsid:ipni.org:names:60476868-2

Figure 1

#### Diagnosis.

Like *Solanumamericanum* Mill., but differing in its glandular pubescence on all vegetative parts, larger flowers with longer anthers, glabrous adaxial calyx lobe surfaces and spreading to appressed calyx lobes in fruit.

#### Type.

Brazil. Bahia: Mun. Maracajú, Lagoa Itaparica 10 km W of São Inacio-Xique-Xique road at the turning 13.1 km N of São Inacio, 300–400 m alt., 26 Feb 1977, *R.M. Harley [with S.J. Mayo, R.M. Storr & T.S. Santos] 19125* (holotype: RB [RB00464327, acc. # 271981]; isotype: CEPEC [acc. # 19367]).

**Figure 1.**
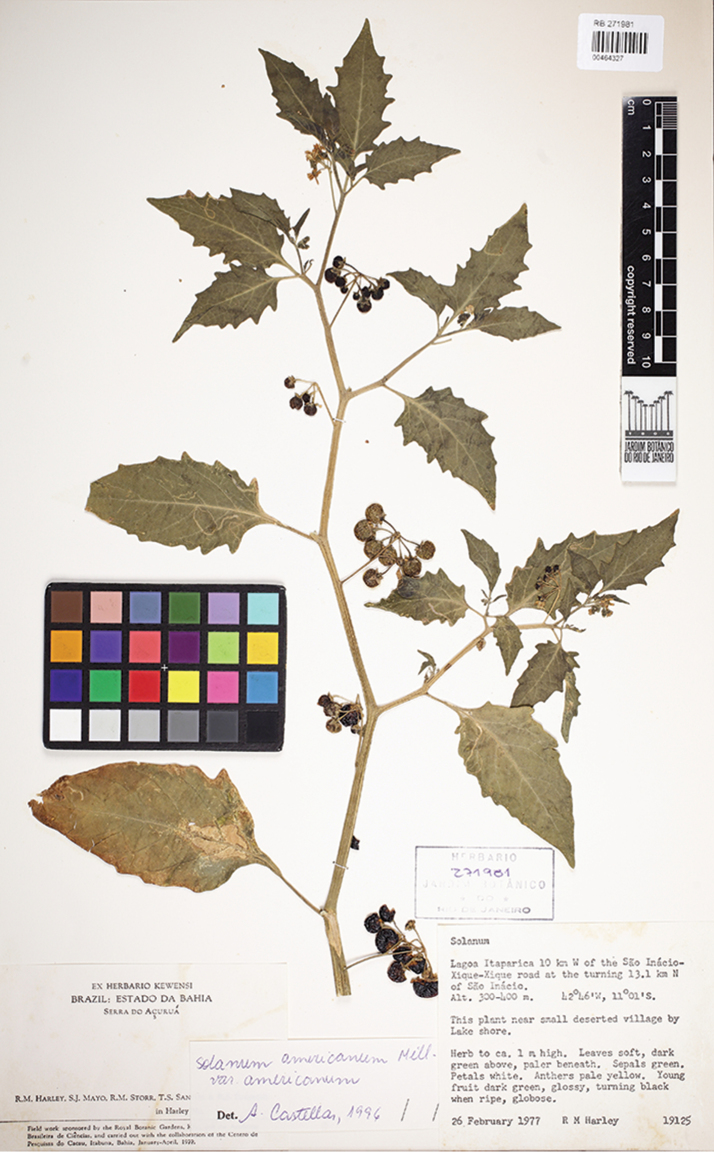
Photograph of the holotype of *Solanumcaatingae* (*Harley et al. 19125*, RB). Image courtesy of the Jardim Botânico do Rio de Janeiro (JBRJ).

#### Description.

Perennial herb, 0.4–1 m tall, perhaps occasionally annual or only persisting for a few years. Stems terete or slightly angled, lacking spinescent processes; young stems densely to sparsely pubescent with spreading glandular, simple uniseriate trichomes 0.5–1 mm long, the trichomes 4–15 celled, drying translucent; new growth densely glandular pubescent; bark of older stems greenish-brown or pale tan. Sympodial units unifoliate or difoliate, the leaves not geminate. Leaves simple, shallowly toothed, 2.5–10 cm long, 1–4.5 cm wide, ovate to broadly elliptic, widest in the lower half, membranous; adaxial and abaxial surfaces evenly glandular-pubescent with simple uniseriate trichomes to 2 mm long, these denser abaxially and along the veins, densely pubescent with minute glandular papillae on both leaf surfaces especially in young leaves; principal veins 4–6 pairs, drying paler than the lamina; base truncate and then abruptly attenuate on to the distal part of the petiole; margins shallowly and irregularly toothed, the teeth ca. 0.5 mm long, rounded at the tips and broadly deltate to semi-circular in outline; apex acuminate, the tip blunt; petiole (0.5) 1–2 cm, only winged from the attenuate leaf base in the distal half to third. Inflorescences internodal, 2–3.5 cm long, subumbelliform with most flowers in the distal portion or spaced ca. 0.5 mm apart, unbranched or furcate, with 5–8 flowers, densely and finely glandular-pubescent like the stems and leaves; peduncle 1.8–3 cm long; pedicels 0.7–0.8 cm long at anthesis, ca. 0.5 mm in diameter at the base, ca. 0.7 mm in diameter at the apex, slender and tapering, densely glandular-pubescent with short uniseriate trichomes and glandular papillae, spreading at anthesis, articulated at the base but the articulation point somewhat swollen and a minute stump that is darker in colour left on the rhachis, this especially visible in fruiting material; pedicels scars closely packed in the distal part of the inflorescence to 0.5 mm apart, with the lowermost ca. 1 mm distant from the rest. Buds globose to broadly ellipsoid, the corolla strongly exserted from the calyx tube before anthesis. Flowers 5-merous, all perfect. Calyx tube 1–1.5 mm long, conical to broadly conical, the lobes 1–1.5 mm long, ca. 1 mm wide, deltate and spathulate, densely glandular-pubescent like the pedicels with uniseriate trichomes and papillae, the tips rounded. Corolla 0.6–0.9 cm in diameter, white with a darker (green?) central star, stellate, lobed 2/3–3/4 of the way to the base, the lobes 2.5–3.5 mm long, 1.5–3 mm wide, triangular, reflexed to spreading at anthesis, the abaxial surfaces glabrous to sparsely papillate with a few glandular trichomes ca. 0.2 mm long. Stamens equal; filament tube minute; free portion of the filaments 0.5–1 mm long, glabrous or sparsely pubescent with a few weak tangled simple uniseriate trichomes adaxially at the very base; anthers 1.8–2.2 mm long, 0.7–1 mm wide, ellipsoid, bright yellow, smooth, poricidal at the tips, the pores elongating to slits with age. Ovary conical, glabrous; style 3.5–4 mm long, sparsely glandular pubescent with weak tangled trichomes and papillae in the basal half where included in the anther cone; stigma minutely capitate, densely papillate, not markedly different from the style. Fruit a globose berry, 0.7–1 cm in diameter, green when young, maturing shiny black; the pericarp thin but not translucent when dry (drying black); fruiting pedicels 0.9–1.2 mm long, tapering from a base ca. 1 mm in diameter to an apex 1–1.2 mm in diameter, not distinctly woody, spreading and becoming deflexed at fruit maturity, remaining on inflorescence; fruiting calyx not accrescent, the tube 1–1.5 mm long, the lobes 2–2.5 mm long, spreading and later reflexed, covering the lower ca. 1/4 of the berry, the abaxial surfaces not densely papillate (different to *S.americanum* where the surfaces are densely papillate). Seeds (30)50–80 per berry, 1–1.5 mm long, 1–1.2 mm wide, tear-drop shaped with a subapical hilum, reddish-gold, the surfaces minutely pitted, the testal cells pentagonal. Stone cells absent. Chromosome number: Not known.

#### Distribution

**Figure 2.**
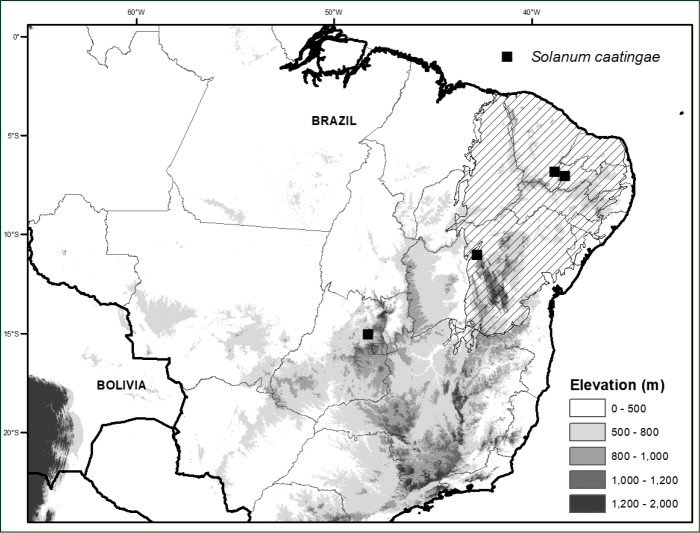
Distribution of *Solanumcaatingae*. Hatched area indicates the Caatinga Biogeographic Domain (sensu IBGE 2004).

(Figure 2). *Solanumcaatingae* is endemic to Brazil; widely scattered collections are known from the states of Bahia, Ceará, Paraiba and Goiás.

#### Ecology and habitat.

*Solanumcaatingae* grows in dry formations known as “caatinga” or “savana estépica” (Eiten 1983; Prado 2003), between 300 and 400 m elevation. The caatinga (from the Tupi language words “caa” forest and “tinga” white) formation is a xerophytic thorn scrub/forest with marked differences in rainfall between wet and dry seasons. The biome occurs within the Caatinga Biogeographic Domain (IBGE 2004) in north-eastern Brazil. The Caatinga Domain is a complex mosaic of many biomes, ranging from the thorn forests of the caatinga proper (see Andrade-Lima 1981) to gallery forest, to humid forests on higher elevations (“brejos de altitude”) and cerrado savannas (Andrade-Lima 1981; Lleras 1997). Like many other morelloid species, *S.caatingae* apparently grows in somewhat disturbed and moist areas within the broader more xerophytic habitat and details of its ecological preferences will remain somewhat unclear until more field observations and collections can be made. All specimens seen were in both flower and fruit, so it is likely to flower and fruit all year round or at least when water is available.

#### Etymology.

The species epithet is a genitive noun and comes from the caatinga vegetation formation (e.g. Olson et al. 2000; IBGE 2004) from where most of the collections of this species are known.

#### Preliminary conservation status

(IUCN 2017). DD (Data Deficient; EOO=55,971 km^2^ [LC]; AOO=16 km^2^ [EN]). The paucity of collections of *S.caatingae* means we cannot assign a preliminary conservation status with any certainty. The widely scattered nature of collections, coupled with the extreme threats to caatinga vegetation, mean that the species is possibly of conservation concern, despite its relatively large EOO. More collections may be hiding in the many sheets of the widespread and common *S.americanum* held in Brazilian herbaria and we hope that the description of this taxon will stimulate its future discovery both in the field and in herbaria.

#### Discussion.

*Solanumcaatingae* is morphologically most similar to the widespread circumtropical weed *S.americanum*. It differs from it most strikingly in its spreading glandular pubescence of translucent trichomes (versus appressed eglandular pubescence of white trichomes) and longer anthers (ca. 2 mm long versus ca. 1.5 mm long). Several other glandular pubescent species of herbaceous solanums occur in the dry forests of South America, but these are mostly from the Chaco biome and do not overlap in distribution with *S.caatingae* (see Särkinen and Knapp 2016). *Solanumcaatingae* can, however, be distinguished from these species (e.g. *S.michaelis* Särkinen & S.Knapp, *S.nitidibaccatum* Bitter, *S.physalifolium* Rusby, *S.sarrachoides* Sendtn., *S.tweedianum* Hook. and *S.woodii* Särkinen & S.Knapp) by its calyx that is not accrescent in fruit with the lobes spreading or slightly reflexed and its shiny black berries with no stone cells. *Solanumarenicola* Särkinen & P.Gonzáles is another glandular pubescent species with which *S.caatingae* could potentially be confused, but that taxon is Amazonian and occurs along rivers in the tropical rainforest; it has not yet been recorded for Brazil, but we expect it to occur in the western part of the country along the border with Bolivia and Peru. Morphologically, *S.arenicola* differs from *S.caatingae* in its larger flowers (8–12 mm in diameter versus 6–9 mm in diameter in *S.caatingae*), longer anthers (3–4 x 0.8–0.9 mm versus 1.8–2.2 x 0.7–1 mm), smaller berry (3.5–7 mm versus 7–10 mm in diameter) and presence of stone cells in the berries.

Several species of European and African polyploid morelloids (e.g. *S.nigrum* L., *S.retroflexum* Dunal, *S.villosum* Mill.) are polymorphic for presence or absence of glandular trichomes and their occurrence does not correlate with relationships based on phenetic studies with molecular markers (Manoko 2007; see Särkinen et al. 2018 for a discussion). In the Americas, however, glandular pubescence is correlated with other characters such as anther length and stone cell presence or absence, suggesting it can be of taxonomic significance.

The type collection *(Harley et al. 19125*) comes from near the edge of the Caatinga Biogeographic Domain as defined by the Instituto Brasileiro de Geografia e Estatística (IBGE 2004) in a highly heterogeneous mosaic of caatinga and cerrado around a seasonal lake (Lagoa Itaparica) with stands of carnaúba palm (*Coperniciaprunifera* [Mill.] H.Moore, Arecaceae). The plant itself was found near abandoned houses in a weedy area with *Waltheriarotundifolia* Schrank (Malvaceae), *Sidaspinosa* L. (Malvaceae) and *Calotropisprocera* (Aiton) W.T.Aiton (Apocynaceae) (R. Harley, in litt., 31 May 2018, extract from field diary dated 26 Feb 1977), suggesting that, like many other morelloid species, *S.caatingae* grows in disturbed sites with at least some moisture, perhaps accounting for its sparse distribution across its range. A duplicate of Harley’s collection was not found in the herbarium at Kew. The specimen collected by Johann Pohl at “Rio Maranhao” was collected between 1817 and 1821 and is from a small tributary of the Rio Tocantins slightly to the north of the Distrito Federal. This is at the very southern edge of the Caatinga Domain and is one of the priority areas for both conservation and study (Tabarelli and Cardoso da Silva 2003).

#### Specimens examined

**(paratypes)**. **BRAZIL**. Ceará: Mun. Lavras de Mangabeira, area a ca. 12 km a N do Distrito de Felixardo, 299 m, 24 Jul 2014, *A. Costa-Lima et al. 1406* (HUEFS, RB). Goiás: Rio Maranhão, sin.dat., *J.B.E. Pohl 2393* (W). Paraiba: Mun. Carrapateira, Sitio Volta, nos arredores do Açude Volta, 404 m, 24 Sep 2014, *A. Costa-Lima et al. 1862* (HUEFS, RB).

### Key to the Brazilian species of black nightshades (Morelloid clade)

*Solanumarenicola* is included here although it has not yet been recorded for Brazil; the species is known from adjacent Peru and Bolivia in lowland Amazonian rainforest.

**Table:** 

1	Plants with simple glandular pubescence on stems and leaves	**2**
–	Plants with simple eglandular trichomes (without glandular tips) on stems and leaves	**4**
2	Calyx markedly accrescent in fruit, covering more than half of the berry; flower buds completely enclosed within the calyx lobes; rare annuals of coastal habitats in southern Brazil (mostly known from Argentina)	***Solanumsarrachoides* Sendtn.**
–	Calyx not markedly accrescent in fruit, the lobes spreading and only covering ca. 1/4 of the berry; flower buds strongly exerted from the calyx lobes; perennials (annuals?) of dry areas within the Caatinga Domain in north-eastern Brazil or in moist Amazonian lowland rain forest in western Brazil	3
3	Anthers 1.8–2.2 mm long; calyx lobes deltate-rounded with rounded apices; mature berry shiny black or purplish-black, without stone cells; in dry areas within the Caatinga Domain in north-eastern Brazil	***Solanumcaatingae* S.Knapp & Särkinen**
–	Anthers 3–4 mm long; calyx lobes long-triangular with acuminate apices; mature berry matte purple-black, with stone cells; in moist Amazonian lowland rain forest probably in western Brazil (not yet recorded)	***Solanumarenicola* Särkinen & P.Gonzáles**
4	Anthers 0.8–2.5 mm long	**5**
–	Anthers (2.5-) 3.5–5.5 mm long	**6**
5	Anthers 0.8–1.5 mm long; fruiting pedicels spreading, not recurved or reflexed; calyx lobes in fruit ca. 1 mm long, strongly reflexed; fruits drop off without pedicels and calyx, leaving behind peduncles with pedicels and calyces still attached; berries shiny black, with 0–4 stone cells; widespread weed	***Solanumamericanum* Mill.**
–	Anthers 1.5–2.5 mm long; fruiting pedicels recurved; calyx lobes in fruit 1.5–3.0 mm long, appressed to the berry, not reflexed; pedicels drop off with fruit, leaving peduncles behind; berries matte black, with no stone cells; in littoral from Santa Catarina to Ceará, less commonly inland	***Solanumchenopodioides* Lam.**
6	Leaf bases truncate; leaves broadly delate to ovate; inflorescence unbranched (rarely furcate); calyx lobes in fruit ca. 1 mm long; lowlands in southern Brazil, from inland or along Amazonian rivers	***Solanumpilcomayense* Morong**
–	Leaf bases attenuate, often decurrent on the petiole; leaves lanceolate, elliptic or more rarely ovate; inflorescence branched or less commonly unbranched; calyx lobes in fruit >1 mm long; Amazonian or southern Brazil	**7**
7	Calyx lobes long triangular with acuminate apices; corolla lobes long and narrow; buds more than 2 times longer than wide, narrowly ellipsoid; plants of lowland Amazonia probably occurring in western Brazil (not yet recorded)	***Solanumarenicola* Särkinen & P.Gonzáles**
–	Calyx lobes deltate or triangular with acute apices; corolla lobes deltate; buds ca. 2 times longer than wide, ellipsoid; plants of south-eastern Brazil	**8**
8	Anthers (2.5-) 3.5–4.5 mm long; pedicels tightly to loosely spaced, recurving/reflexed in most specimens and fruiting inflorescences appearing secund; calyx lobes in fruit 1.5–2 mm long, appressed to the berry; corolla with a dark purple eye, lobed nearly to the base, the lobes strongly reflexed at anthesis; berries with 2 small stone cells; 0–1,700 (-2,300) m elevation in southern Brazil	***Solanumpaucidens* Bitter**
–	Anthers 4.4–5.5 mm long; pedicels arising closely together yet regularly spaced 1 mm apart, spreading in fruit; calyx lobes in fruit 1.5–2.5 mm long, slightly spreading; corolla with a yellow eye, lobed to 1/2–2/3 to the base, the lobes spreading at anthesis; berries without stone cells; above 2,000 m elevation in coastal mountains of Minas Gerais, Rio de Janeiro and São Paulo	***Solanumenantiophyllanthum* Bitter**

## Supplementary Material

XML Treatment for Solanum caatingae

## Supplementary materials

Supplementary material 1 Specimen data for species of the Morelloid Clade occurring in Brazil Data type: occurrence Explanation note: CSV file of specimen and locality data for morelloid species (black nightshades) occurring in Brazil. These data are also deposited as a dataset on the Natural History Museum Data Portal (https://doi.org/10.5519/0034287). This is an open access article distributed under the terms of the Creative Commons Attribution License (CC BY 4.0), which permits unrestricted use, distribution, and reproduction in any medium, provided the original author and source are credited.Sandra Knapp, Tiina Särkinen
